# Editorial: Key Electrochemical Energy Reactions Catalyzed by Nanomaterials

**DOI:** 10.3389/fchem.2019.00881

**Published:** 2019-12-20

**Authors:** Tianyi Ma, Lei Zhang, Zhenhai Wen

**Affiliations:** ^1^Discipline of Chemistry, University of Newcastle, Callaghan, NSW, Australia; ^2^School of Chemistry and Chemical Engineering, South China University of Technology, Guangzhou, China; ^3^Fujian Institute of Research on the Structure of Matter, Chinese Academy of Sciences, Fuzhou, China

**Keywords:** renewable energy, electrocatalysis, photocatalysis, nanomaterials, surface chemistry

The application for (photo)electrochemical technologies is set to expand rapidly in the recent several decades as demand grows in the environment protection, clean energy storage, and supply. Several unique characteristics of functionalized nanostructured materials or transition metal-nitrogen-carbon complex make them ideal catalyst candidates for combining high energy and power at the material level. These advantages include: (i) increased photo/electrochemically active surface areas for charge transfer, (ii) reduction of photo/electronic and ionic transport resistance at smaller diffusion length scales, and (iii) the ability to incorporate high-energy materials into a nanostructured framework capable of sustaining high powers (Lee et al., 2011; Seh et al., 2017; Sagar et al., 2018).

Recent advancement of nanostructured materials or transition metal-nitrogen-carbon complex has improved (photo)electrochemical performance significantly, especially in term of catalysis, including but not limited to oxygen reduction reaction (ORR), oxygen evolution reaction (OER), hydrogen evolution reaction (HER), nitrogen reduction reaction (NRR), and carbon dioxide reduction reaction (CRR). Among them, the noble metal-based catalysts exhibit attractive catalytic performance, but their rare nature hinders large-scale application. In recent years, a large variety of non-noble-metal or non-metal-based alternative catalysts using abundant and low-cost 3d metals (e.g., Fe, Co) has been studied with increasing activity and stability (Yan et al., 2019).

As future applications, besides considering the low cost, a major challenge will be to reduce and bridge the performance gap by combining high specific surface active sites, high photo/electronic conductivity and good mechanical and chemical stability. In this Research Topic, we present a highlighted collection of original research articles that show how transition metal-nitrogen-carbon complex and nanomaterials such as 2D transition metal nanofilms, hollow metal microspheres, and graphene offer opportunities to develop potentially novel material structures that can reach these goals.

Recently, it has been indicated that developing cheap, highly efficient, and stable bifunctional electrocatalysts for both hydrogen and oxygen evolution reactions (HER and OER) has garnered a great interest with studies showing their encouraging large-scale application of water splitting technology. Zhang et al. reported novel CoS_2_ nanoparticles supported on nitrogen-doped graphene (CoS_2_@N-GN) by a one-step hydrothermal method; the resultant hybrid exhibited remarkable overall electrocatalytic activity toward OER and HER in the alkaline electrolyte with enhanced long-term stability. Moreover, attributed to the high intrinsic activity of CoS_2_ nanocrystals, efficient electron transfer provided by N-doped graphene and the synergetic coupling interaction between two components, the CoS_2_@N-GN enabled the assembled water splitting device with low cell voltage, high efficiency, and prolonged operational life, which would provide insight into the rational design of transition metal chalcogenides for highly efficient and durable hydrogen and oxygen-involved electrocatalysis. On the other hand, implanting noble metal onto transition metal-nanofilms as one of the significant strategies has been shown to greatly control and increase the catalytic activity toward the ORR performance. More specifically, Pd-based electrocatalysts show excellent stability in alkaline solution owing to a less corrosive environment, while Co-decorated catalysts have the advantages of their low cost and promising applications in oxygen electrode (Cheng et al., 2017a,b). As a demonstration, Pd nanoparticles implanted onto Co nanofilms (Pdx/Co-nanofilms/C) were synthesized on an immiscible ionic liquid (IL)/water interface by An et al. Due to a marked distortion of crystal lattice and surface roughness showing their larger catalytic areas, the Pdx/Co-nanofilms/C catalysts exhibited enhanced ORR catalytic activity in both acid and alkaline media. In conjunction, the designed catalysts exhibited superior stability in alkaline media after using a proper heat-treatment method. Besides, with the advantages of alkaline fuel cells increasingly prominent due to the emergence, development and application of anion exchange membranes, ORR is also an important part of the alkaline fuel cell (Lefevre et al., 2009; Wang et al., 2012; Shen et al., 2018). Among the non-precious metal catalysts, the transition metal-nitrogen-carbon complex (M-N-C) as the most attractive candidate, has achieved landmark achievements and might be commercially employed in fuel cells in the near future (Artyushkova et al., 2015; Kim et al., 2017; Li et al., 2017). Thus, Gu et al. showed a nitrogen-rich ligand PIPhen as a precursor that was reacted with Fe^2+^ to form the coordination polymer on the carbon powder (Fe-PIPhen/C). The prepared Fe-PIPhen/C catalyst possessed high ORR activity, only slightly lower than that of the Pt/C catalyst. Without employing the pyrolysis process, the Fe-PIPhen/C catalyst has advantages in low cost and controllable structures, being promising alternatives to the Pt/C catalyst in the fuel cell ORR process.

Although nanostructured materials with a variety of useful functionalities are widely applied in electrocatalysis and energy storage (Guo et al., 2017), some environmental-friendly magnetic materials as important photocatalysts are also potential for a wide range of practical applications (Ma et al., 2018), such as hematite (α-Fe_2_O_3_). Since self-assembled hematite with highly specific hollow nano/micro-structures and unique properties have emerged as being of great interest to material scientists, porous self-assembled α-Fe_2_O_3_ hollow microspheres were successfully synthesized by Yin et al. via a simple and feasible IL-assisted solvothermal method. In this work, the IL [C_4_Mim]BF_4_ was used not only as solvents, but also as templates for the formation of porous hollow spheres with improved properties (Endres et al., 2003; Cooper et al., 2004; Liu et al., 2006). Attributed to the self-assembled structure and higher surface area, the as-synthesized α-Fe_2_O_3_ hollow spheres exhibited excellent photocatalytic activity in Rhodamine B (RhB) photodegradation. Additionally, the main formation mechanism of the porous hollow structures has been proved to be a nucleation–aggregation evacuation; and the as-prepared photocatalysts can be recycled easily due to the ferromagnetic properties. As knowing that the high recombination rates of photogenerated electron-holes inhibit the catalytic activity of semiconductor photocatalyst, Wu et al. introduced a simple hydrothermal method to synthesize the heterojunctions of flower-like g-C_3_N_4_/BiOBr composites as photocatalysts. BiOBr-g-C_3_N_4_ (4:1) showed the most excellent properties of photocatalytic degradation bisphenol (BPA) under the visible light due to its narrower bandgap than that of pure BiOBr. Moreover, the separation of photogenerated carriers can be facilitated by the heterostructure between BiOBr and g-C_3_N_4_.

We believe that the Key Electrochemical Energy Reactions Catalyzed by Nanomaterials Research Topic exemplifies various nanomaterials research development in the application of (photo)electrocatalysis and provides research strategies on how to improve these catalytic performances. The (photo)electrocatalysis plays a central role in the environment, clean energy conversion, enabling sustainable processes for future technologies (Seh et al., 2017). Thus, the Research Topic aims to know the research frontier of Energy Reactions Catalysts, with faster and more efficient methods to capture this understanding from these more advanced researches, which can further guide the community to seek for high-performance catalysts.

## Author Contributions

All authors listed have made a substantial, direct and intellectual contribution to the work, and approved it for publication.

### Conflict of Interest

The authors declare that the research was conducted in the absence of any commercial or financial relationships that could be construed as a potential conflict of interest.
